# Analyzing Self-Explanations in Mathematics: Gestures and Written Notes Do Matter

**DOI:** 10.3389/fpsyg.2020.513758

**Published:** 2020-11-23

**Authors:** Alexander Salle

**Affiliations:** Institute for Mathematics, School of Mathematics/Computer Science, Osnabrück University, Osnabrück, Germany

**Keywords:** self-explanation, gesture, multimodality, trigonometry, complex numbers

## Abstract

When learners self-explain, they try to make sense of new information. Although research has shown that bodily actions and written notes are an important part of learning, previous analyses of self-explanations rarely take into account written and non-verbal data produced spontaneously. In this paper, the extent to which interpretations of self-explanations are influenced by the systematic consideration of such data is investigated. The video recordings of 33 undergraduate students, who learned with worked-out examples dealing with complex numbers, were categorized successively including three different data bases: (a) verbal data, (b) verbal and written data, and (c) verbal, written and non-verbal data. Results reveal that including written data (notes) and non-verbal data (gestures and actions) leads to a more accurate analysis of self-explanations than an analysis solely based on verbal data. This influence is even stronger for the categorization of self-explanations as adequate or inadequate.

## Introduction

Imagine a learner considering a worked-out example that presents the solution of a task dealing with right-angled triangles. The given example includes the complete solution of that task without describing the theorems or principles that were used for the calculations depicted. While reading the worked-out example, the learner thinks aloud: “*Ok, this triangle ABC*,… *the missing side*… *was calculated. And they made it*… *the Pythagorean Theorem, I think. This side here*… *then would be*… *uhm, square root of. yes, that works.*” When confronted with worked-out examples, texts or other instructional material, learners can learn in different ways with worked-out examples, e.g. superficially or thoroughly. The statement above illustrates an advantageous learning approach: The learner explains to herself the calculations in the material drawing on her (activated) prior knowledge of the Pythagorean Theorem, she is *self-explaining*.

Self-explaining means explaining something to oneself by generating information not provided in the given material and by creating inferences to organize given or new information in order to make sense of the material – it is a generative activity that occurs during learning (Chi, 2000, 2009; Fiorella and Mayer, 2016). Typical examples of self-explaining are activating and integrating prior knowledge, integrating different representations in a text, and clarifying requirements for depicted mathematical operations (Chi et al., 1989; Chi, 2000). To distinguish cognitive processes and overt activities of learners, utterances generated during self-explaining—like the statement depicted above—are called *self-explanations* (Chi, 2000, 2009).

Self-explaining as a learning strategy can serve to aid learners’ comprehension of a topic (de Koning et al., 2011; Chiu and Chi, 2014; Wylie and Chi, 2014; Fiorella and Mayer, 2016). There are several broadly approved methods to foster self-explaining like different forms of self-explanation prompts (e.g. Chi et al., 1994; Berthold et al., 2009; van der Meij and de Jong, 2011; Hefter et al., 2015) or trainings (McNamara, 2004; Kurby et al., 2012; Hodds et al., 2014), and it has been shown that these methods increase learning outcomes and understanding. In summary, when learning with instructional material, self-explaining is essential to a deep and meaningful understanding (Renkl, 2014; Wylie and Chi, 2014). Self-explanations, quantified and counted based on transcripts or recordings, have been identified as main predictors for learning outcomes in psychometrical designs when investigating the benefits of instructional material, e.g. worked-out examples or instructional texts (e.g. Chi et al., 1989; Butcher, 2006; Griffin et al., 2008).

These findings have been replicated in different domains. Especially, a plethora of studies focusing on instructional material and self-explanations has been conducted with mathematical or mathematically-related content (e.g. Chi et al., 1989; Renkl, 1997; Neuman and Schwarz, 1998; Stark, 1999; Renkl et al., 2004).

Until the 1980s, many researchers thought of mathematics as a purely cognitive and disembodied discipline, “based on the premise of a human mind-body split and of the transcendence of mind over body” (Gerofsky, 2014). Since then, many studies have shown that the whole body and its different modalities are important parts of the communication about and the learning of mathematics (Núñez et al., 1999; Arzarello et al., 2008; Radford, 2009; Gerofsky, 2010).

While there are numerous results of qualitative as well as quantitative studies underlining this importance, it remains unclear so far, how significant the inclusion of further modalities for quantifications of learning processes really is. In how far do individual differences, e.g. in gesturing behavior, influence the quantification of learning processes? Apart from the methodological level, these questions are also relevant when estimating privacy issues (audio recordings vs. video recordings) and necessary resources (duration of data analysis, expensive digital tools for data analysis) for empirical studies. Since quantifying self-explanations is an important and frequently used approach in studies analyzing meaningful learning, self-explaining is a fruitful concept for further investigations. Previous research on self-explanations mostly rely solely on verbal transcripts of think-aloud sessions (Ericsson and Simon, 1993; Chi, 1997) and exclude non-verbal or written data. In most cases, thinking aloud in self-explanation studies refers to the recording of verbal utterances, (i) without giving learners the opportunity to take notes, respectively, without considering written notes or their use during the learning sessions, and (ii) without considering gestures, actions or other non-verbal data during analysis.

The main question this paper intends to answer can, therefore, be stated as follows: *In what way does considering written notes and gestures influence the analysis of self-explaining in mathematical learning processes?*

Pursuing this question, an empirical study was conducted in which undergraduate students performed a learning exercise with worked-out examples from the field of complex numbers. Their learning processes were videotaped and categorized based on different data sources in order to analyze the influence of non-verbal and written data on the reconstruction of learning processes and self-explanations. Besides the results for self-explanations in particular, the design and the data analysis in this paper may be a transferable example for investigations that perform quantitative analyses to show the importance of gestures and notes in learning processes.

## Literature Review

### Self-Explanations

Adequate use of cognitive strategies and engaging in domain specific cognitive activities are essential to meaningful and deep learning (Swing and Peterson, 1988; Murayama et al., 2012). Since the seminal work of Chi et al. (1989), a large number of empirical works has shown the analytic and predictive power of self-explanations, which constitute a class of meaningful cognitive activities. Self-explanations are the main predictors for learning outcomes when investigating the benefits of instructional material like worked-out examples or instructional texts, and facilitate evaluation of instructional methods (e.g. Chi et al., 1989; Renkl, 1997, 2005; Butcher, 2006; de Koning et al., 2011).

Although both self-explaining and explaining are constructive activities (Chi, 2009), there are clear differences. While self-explaining is a cognitive activity that does not require verbalization (although it can be traced through overt activities and verbalization), explaining is inherently bound to communication. Self-explaining is based on one’s own prior knowledge, whereas explaining must be based on the knowledge of another person. From a cognitive point of view, explaining needs additional selecting and organizing processes to give others a suitable and comprehensible explanation (Fiorella and Mayer, 2016). Typical examples of self-explanations are inferencing from depicted data (Wong et al., 2002), repairing misconceptions (Chi, 2000), explaining solution steps with prior knowledge (Chi et al., 1989), explaining the goals of an operation (Chi et al., 1989; Renkl, 1997; Renkl et al., 1998), and integrating symbolic calculations and iconic representations (Aleven and Koedinger, 2002). By definition, self-explanations can be incomplete, fragmented or even wrong, e.g. explaining the goals of an operation could address the wrong goals (Chi, 2000).

Self-explanation studies apply experiments in different domains, covering topics like the blood flow and the circulatory system (e.g. Ainsworth and Loizou, 2003; de Koning et al., 2011) or LISP programming (Pirolli and Recker, 1994; Bielaczyc et al., 1995). However, one of the domains most often investigated is mathematics. Such studies often deal with mathematical or mathematically-related content on a lower secondary level like elementary probability theory (e.g. Renkl, 1997; Renkl et al., 2004), compound-interest calculations (e.g. Renkl et al., 1998), algebra word problems (e.g. Neuman and Schwarz, 1998; Neuman et al., 2000), elementary geometry (Wong et al., 2002), and Newton’s laws and calculating forces (e.g. Chi et al., 1989; Chi and VanLehn, 1991). To determine the impact of self-explaining on test performance in those domains quantitatively, self-explanations are categorized and *quantified* based on data that is typically collected in one of two common procedures:^1^

(1) Coding of written texts produced by learners after or during their work with learning materials or tasks (e.g. Schworm and Renkl, 2006; Berthold et al., 2009);
(2) Coding of protocols from think-aloud sessions that are recorded during or after learners’ work with instructional material or tasks (e.g. Chi et al., 1989; Renkl, 1997; Durkin and Rittle-Johnson, 2012; McEldoon et al., 2013).

In the first case, the participants themselves sum up their learning processes in writing. The resulting products are examined for passages which can be identified as self-explanations. In contrast to the second case, this procedure includes all available data (the written text).

During the second procedure, the participants, working individually, are instructed to think aloud while learning and working with different materials and/or tasks. Typically, the procedure follows the methodological principles of Ericsson and Simon (1993).

In the studies following the second procedure, participants in thinking aloud settings studies were audio- or videotaped. Subsequently, the recorded data was transformed into verbal protocols for further coding procedures.^2^ When video data was collected, it was not considered in the analyses of verbal protocols (e.g. Bielaczyc et al., 1995; Neuman et al., 2000). If participants were allowed to take notes or sketch diagrams, these written documents were not analyzed synchronously with the verbal protocols and oftentimes not analyzed at all.^3^

Some authors try to gain insight into the role of incorrect self-explanations for learning (Wilkin, 1997; McNamara, 2004; Butcher, 2006; Ainsworth and Burcham, 2007; de Koning et al., 2011). Both negative and positive effects of incorrect self-explanations are revealed: For less demanding activities such as paraphrasing, the number of incorrect self-explanations correlates negatively with subsequently measured performance; for more demanding activities such as inferencing new information based on given texts, however, positive correlations show the potential benefit of incorrect self-explanations (McNamara, 2004). These findings correspond with results that show the learning potential of incorrect self-explanations (Chi, 2000). However, the majority of studies on self-explanation do not distinguish between correct and incorrect self-explanations, although from a psychological as well as from a domain-specific perspective the differentiation would be an important issue for investigations of learning processes across all domains, especially for mathematics.

### Learning and Multimodality

Many studies in the last 30 years have demonstrated that the whole body and its modalities are an important partaker of and a constitutive entity for communication and learning (Lakoff and Johnson, 1999; Núñez and Freeman, 1999; Gerofsky, 2014). Therefore, thoughts and language are created and expressed through many “modalities linked together – sight, hearing, touch, motor-actions, and so on.” (Gallese and Lakoff, 2005, p. 456). Although especially mathematics is typically regarded as a highly cognitive discipline, many researchers have (re-)discovered and verified the body’s importance for mathematical learning (Núñez et al., 1999; Lakoff and Núñez, 2000; Gerofsky, 2014). By emphasizing the importance of bodily modalities and their role for the origination of mathematics, gesturing can be seen as “a key element in communication and conceptualization” (Radford et al., 2009, p. 93).

As an important part of a multimodal perspective, analyses have shown teachers and learners gesturing frequently and intensely when communicating and thinking about mathematics (e.g. Alibali and DiRusso, 1999; Goldin-Meadow and Singer, 2003; Greiffenhagen and Sharrock, 2005; Edwards, 2008; Radford, 2009; Yoon et al., 2011; Kita et al., 2017). Gesturing can support uttered words as well as supplement or contradict them in different ways (e.g. Alibali and Goldin-Meadow, 1993; Alibali and DiRusso, 1999; Goldin-Meadow and Singer, 2003; Hegarty et al., 2005; Robutti, 2006; Arzarello et al., 2008). In combination with notes or other inscriptions, gestures are applied in specific and subtle ways to construct and communicate mathematical knowledge (Krause, 2016; Krause and Salle, 2016, and, more general, Streeck and Kallmeyer, 2001).

Some studies analyze the use of gestures during explanations or think-aloud settings. When learners explain things to each other or to a video camera, their expressions go beyond verbal utterances and are often accompanied by different kinds of gestures (Schwartz and Black, 1996; Emmorey and Casey, 2001; Hegarty et al., 2005; Salle, 2014). In about 50% of all think-aloud sessions with students who solved gear-problems, Schwartz and Black (1996) found that content-related “rotating” and “ticking” gestures could be observed. Hegarty et al. (2005) report that 98.5% of all identified verb phrases in a gear-problem experiment were accompanied by pointing and tracing gestures, revealing “important individual differences in the use of gesture in both communication and inference” (p. 354). Other works show how gestures are used in explanations to depict certain aspects of verbalized parts (Koschmann and LeBaron, 2002; Alibali and Nathan, 2012).

Findings about the role of different modalities in self-explanation analyses are rare. In eye-tracking studies, learners’ direction of gaze while integrating information given in material was analyzed; including data from eye-tracking devices allows for more accurate analyses of self-explanation (Merten, 2002; Conati and Merten, 2007; She and Chen, 2009; Hodds et al., 2014). However, no systematical analysis of self-explanations and the role of bodily modalities and inscriptions in think-aloud settings has been carried out yet. Thus, the extent to which the consideration of spontaneously produced written notes and non-verbal utterances, such as gestures, could influence the identification of self-explanations in think-aloud settings remains unclear. A multimodal analysis could help to identify adequate and inadequate self-explanations, improve explanations for learning gains or optimize the design of learning materials by providing better measures of self-explanation.

## Conceptual Framework and Research Questions

### Multimodality and Self-Explanations

An utterance is understood as an expressive product in the sense of the multimodal framework of Edwards and Robutti (2014). Hence, expressive products are “physical ‘traces,’ whether permanent or ephemeral, of people’s actions” (p. 13). That includes speech and gestures as bodily based expressive products, and inscriptions like written words, symbols, graphs and visuals as external to the body (ibid.).

Applying this definition, spoken words as well as gestures and written products like sentences or drawings can “become fully partakers of the utterance itself” (Nemirovsky and Ferrara, 2008, p. 162). The different expressive products can be seen as facets of one single underlying mental process (Robutti, 2005; Edwards and Robutti, 2014) and, thus, allow identification of specific cognitive processes. Three main types of utterances (expressive products) can be distinguished:

• *Verbal utterances*: spoken sentences and words, shouts and other sounds.
• *Written utterances*: written inscriptions such as characters, words, sentences with specific syntax, drawings, figures, markers.
• *Non-verbal utterances*: gestures, sign language^4^, facial expression, gaze, actions like the movements when writing or drawing.

Based on the remarks above, the definition of a self-explanation can be broadened. In the classical definition, self-explanations are defined as “units of utterances” produced by self-explaining (the cognitive activity), whereby utterances are meant to be verbal (Chi, 2000, p. 165). Hence from a multimodal perspective, a *self-explanation* will refer to *a unit of intertwined (verbal, written, and non-verbal) utterances produced by self-explaining.* This definition was used for the present study. All forms of verbal, written and non-verbal utterances that were recorded on video are considered in this paper except for gaze and facial expression, which were not included in the analysis.

### Gestures

Gestures are bodily based expressive products (Edwards and Robutti, 2014), cognitive processes are mirrored in speech and gesture. Gestures occur in combination with speech, but they also have self-oriented functions that may occur in combination with thought (Alibali et al., 2000; Kita et al., 2017). This paper follows McNeill (1992) and Kita et al. (2017) in their definition of gestures as idiosyncratic spontaneous movements of the hands and arms which depict action, motion, or shape, or indicate location or trajectory, they “include iconic gestures, metaphoric gestures, and deictic gestures” (Kita et al., 2017, p. 245) in the taxonomy described by McNeill (1992) and the differentiation of iconic gestures in mathematics as iconic-physical and iconic-symbolic formulated by Edwards (2008). As long as movement is not part of a functional act (taking notes, measuring something with a ruler), the movement is a gesture; otherwise, it is an action with a purpose (Goldin-Meadow, 2003; Kendon, 2004; Sabena, 2008).

In think-aloud scenarios, gestures can convey important information (Schwartz and Black, 1996; Hegarty et al., 2005; Yammiyavar et al., 2007); they are, therefore, co-thought and/or co-speech gestures. Following the definition of self-explanations from a multimodal perspective, a gesture as well as an action with or without simultaneous speech may allow a coder to identify self-explaining activities.

### Inscriptions

An inscription is defined as “an external ‘representation,’ whether symbolic or imagistic, which is non-ephemeral and therefore amenable to reflection, review, and revision” (Edwards and Robutti, 2014). Since mathematics makes much use of external representations like symbols or graphs, inscriptions play an important role in doing, communicating and learning mathematics (Arzarello et al., 2011; Krause, 2016). Learners create and use inscriptions on paper or other mediums to store and highlight important information for themselves (Kiewra, 1989; Kobayashi, 2005, 2006), to organize them in specific ways (e.g. Eppler, 2006; Kenehan, 2007), or to use such collections when studying (Luo et al., 2016).

During think-aloud procedures, learners can refer to inscriptions already present in the instructional setting or produced by the learners themselves; therefore, researchers may identify self-explaining processes by considering inscriptions in combination with speech and gestures or without them.

### Adequate and Inadequate Self-Explanations

Whether a self-explanation is “correct” or not depends not only on the utterance itself, but also on the content to be learned and the aims of the instructional setting. For example, the notion of a tangent line as a line that touches a circle at one point is absolutely adequate in elementary Euclidean geometry. When it comes to functions and calculus, however, this conception only holds true for special cases and areas. Tangent lines on graphs of third grade polynomials might not fit this explanation.

Such examples illustrate that a classification into “right” or “wrong” self-explanations is difficult. Hence, a classification that distinguishes between *adequate* and *inadequate* self-explanations, always matched to the goals of an intervention and the instructional material itself, fits more precisely and is used throughout the paper.

### Research Questions

Two research questions will guide the following analyses:

(1) Does the consideration of non-verbal utterances (e.g. spontaneous gestures and actions) and written utterances (e.g. notes and diagrams) alter or support the coding of self-explanations?
(2) Does the consideration of non-verbal and written utterances alter or support the determination of self-explanations as adequate or inadequate?

## Materials and Methods

### Participants

The subjects were 33 undergraduate students at a German university (22 females, 11 males) who voluntarily participated in this study. The students ranged in age from 21 to 25 years (*M* = 23.2, *SD* = 1.1), all of them spoke German fluently. All participants were enrolled in teacher training courses for middle school mathematics at the time of the experiment. They were in the third, fourth or fifth semester of their course. All participants were familiar with worked-out examples and computers.

### Materials

The participants worked with three worked-out examples that addressed the multiplication of complex numbers. The chosen topic was new and relevant to them: First, it concerned elementary concepts and objects like polynomials, the fundamental theorem of algebra and trigonometric functions. Dealing with complex numbers helps participants in understanding these contents, which will be relevant for teaching in school, from a more general point of view. Second, this topic represents a foundation for further lectures in algebra, analysis, geometry, etc. Third, the experience of becoming acquainted with a new number system has parallels to school children’s first encounter with rational and real numbers, and, thus, gives future teachers the chance to reflect on certain aspects and obstacles concerning the encounter with new numbers.

**FIGURE 1:**
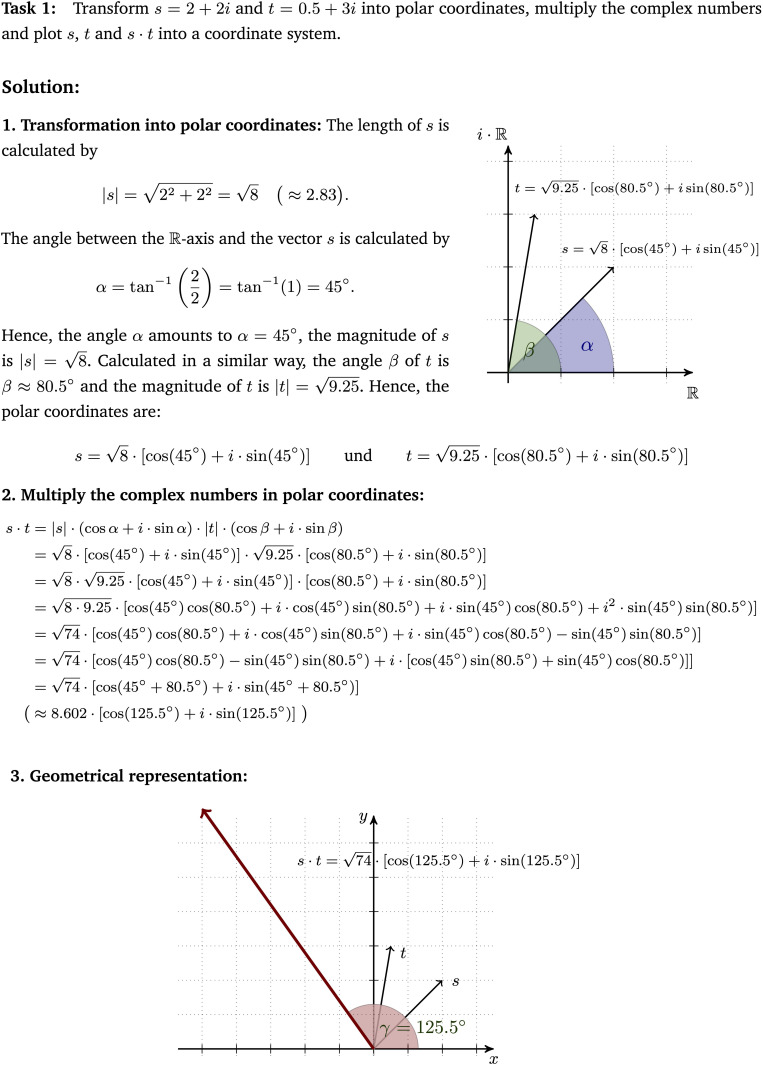
First worked-out example that was used in the intervention phase (translated from the German original, scaled-down version).

Worked-out examples were chosen because they allow a structured investigation of self-explanations and constitute a common format in self-explanation research studies (see literature review). Every used worked-out example (Figure 1) was divided into three parts: (1) transformation of Cartesian coordinates into the trigonometric form of polar coordinates, (2) calculation of the product of two complex numbers represented in the trigonometric form and (3) the geometrical representation of the calculated product.^5^ The second worked-out example showed a second solution to a similar multiplication task and followed the same structure as the first example. In contrast to the first example, the coefficients of the complex numbers were fractions which represented vectors outside the first quadrant of the coordinate system. The third worked-out example dealt with trigonometrically represented polar coordinates that had to be transformed into Cartesian coordinates. Subsequent to this transformation, the Cartesian coordinates were multiplied and geometrically represented. Furthermore, the material contained different representations (symbolic calculations, geometrical representations and a part where those two were intertwined).

### Objects of the Intervention

The following list provides a selection of favored self-explanations likely to arise during learning with the first worked-out example. They depict general principles of and insights into the mathematical topic and were results of a mathematical-content analysis^6^ that was based on Hankel (1867) and Courant and Robbins (2010). The complete list forms the basis for the identification of adequate self-explanations (see Supplementary Material and section “Coding of Self-Explanations”). Numbers in parentheses refer to the three parts of the first example. A learner…

(1) explains the calculation of the vector’s length as application of the Pythagorean Theorem.
(1) recognizes the calculation of the angle α as the application of a trigonometric equation in a right-angled triangle.
(1) integrates the symbolic representation of a complex number and its parts with respective characteristics of the geometrical counterpart.
(2) explains the change of the algebraic sign between line four and five of the calculation by the relation *i*^2^ = −1.
(2) explains the simplification from line six to seven of the calculation as an application of the addition theorems.
(3) identifies the factor $\sqrt{74}$ as the length of the vector *s*⋅*t*.
(3) recognizes that the resulting angle of *s*⋅*t* is the sum of α and β.

### Procedure

First, participants had to complete a pre-test assessing their prior knowledge and competencies concerning complex numbers, trigonometric calculations and functions, and rules of calculating. The test contained 20 items where students had to draw complex numbers in different representations in coordinate systems (4), simplify simple and more complicated symbolic terms with roots and complex numbers (6), determine sine and cosine on right-angled triangles and in the unit circle (6) and give reasons for properties of real and complex numbers (4). The participants had 45 min to complete the test. On the one hand, the results revealed whether participants had the necessary basic arithmetic and algebraic knowledge for the intervention.^7^ On the other hand, participants with too much experience in the field of complex numbers and polar coordinates could possibly be excluded from the study.^8^

In the intervention phase, the participants worked individually with three worked-out examples presented on paper. The assignment given to them was explained in the following way: “Try to understand the worked-out examples. Signal when you have finished. And please think aloud.” After any period of 20 s of silence, the participants were reminded to think aloud. There was no time limit. The participants were permitted to use a prepared ‘cheat sheet’ with definitions and formulas, a pen, a triangle ruler and a calculator application on the computer screen. The think-aloud procedure followed the guidelines of Ericsson and Simon (1993) and Greene et al. (2011). Before the intervention phase there was a short training sequence for the think-aloud procedure. No guidelines on taking notes or gesturing were provided, so all occurrences of gestures and notes were produced spontaneously.

### Data

The data base for the analysis of self-explanations consisted of video recordings from the intervention phases of 33 participants. These intervention sequences contained *verbal data* (participants’ voices) and *non-verbal data* (recordings of the participants’ upper bodies and bodily actions, recorded by the webcam on top of a computer display, and the recordings of calculations made on the computer screen). Furthermore, written notes, comments and calculations made on the worksheets, the ‘cheat sheets’ or additional blank paper were collected (referred to as *written data*).

### Analysis

To analyze the data, a qualitative content analysis consisting of two phases was conducted (Mayring, 2000; Lamnek, 2010). The units of coding, context and analysis were refined successively.

#### Pilot Phase

During the pilot phase, preliminary category schemes were derived from a literature review (see section “Coding of Self-Explanations”). Based on two video sequences of students (unit of coding) who did not participate in the main study, the category schemes were revised, refined and adapted to the empirical findings (Mayring, 2000). Specifically, categories were extended to include written and non-verbal data, e.g. regarding the ways in which special examples of self-explanations were uttered through written or non-verbal expressive products, and regarding their intertwining with verbal utterances. The unit of context comprised a learning session of a participant, and the units of analysis comprised verbal utterances including gestures and notes. All codings were based on semantic features.

#### Main Phase

Data gathered during the main phase consisted of video sequences from 33 participants. The units of context and analysis were identical to those employed during the pilot phase. In order to compare the self-explanations coded on the basis of different data and to follow common frameworks in self-explanation studies, recordings were divided into units of analysis based on verbal data. A unit of analysis was a sentence, a half-sentence or a shorter utterance, separated from other sentences by pauses. The segmentation of the data was done by both coders together. Disagreements were discussed and solved. This division was maintained throughout all coding procedures. The coding procedures are described in the next paragraph.

## Coding of Self-Explanations

The 33 video sequences were coded three times in consecutive coding procedures (described in sections “First Coding Procedure – Verbal Data,” “Second Coding Procedure – Verbal and Written Data,” and “Third Coding Procedure – Verbal, Written and Non-verbal Data”). Using the category schemes resulting from the pilot phase, each coding procedure was applied by a total of two coders familiar with the mathematical content and the research method. These coders coded the video material in all three procedures. 10% of the videos were encoded by both coders to determine the inter-coder reliability (described in section “Inter-Coder Reliability”). The first procedure used only the verbal data from the video recordings to analyze verbal utterances, which included all spoken words, sentences and sounds. During the second procedure, the verbal data from the recordings and the written data were included. During the third procedure, the verbal, written and non-verbal data were included. Facial expressions were omitted from the analysis. The units of analysis were held constant throughout all procedures to allow for a one-to-one comparison between the analyses and thus facilitate quantitative comparisons. Examples of self-explanations identified during each of the three coding procedures can be found in the results section.

### First Coding Procedure – Verbal Data

Based on the division into units of analysis, the verbal data was categorized first. The term “verbal data” refers to the audio track of the video recording, the first coding procedure was carried out based on this audio track. Two decisions were made for every unit of analysis: (a) Can a self-explanation be identified? (Identification of self-explanations). (b) If so, can the self-explanation be identified as adequate or inadequate with respect to the mathematical goals of the intervention? (Determination of adequate and inadequate self-explanations).

(a) *Identification of self-explanations*. Based on the verbal data, it was coded whether a unit of analysis was a self-explanation or not, using the following category scheme:

• (*Self-explanation*): A unit of analysis was coded as a self-explanation if a generation of inferences and/or a mapping of inferences or information onto the learners’ existing mental models could be identified; hence, if self-explaining could be reconstructed. Typical examples are activating prior knowledge for explanations of solution steps, calculations or representations; integrating different representations, e.g. symbolic and geometrical; and drawing inferences from information depicted in examples or the ‘cheat sheet.’
• (*No self-explanation*): Examples for passages coded as no self-explanation are: reading a sentence from the instructional material without signs of bringing in new information through written or non-verbal utterances, or mentioning a number and pointing to it without integration of other information.

Category schemes used to develop the scheme for identification of self-explanations in the present study were those published by Chi et al. (1989), Renkl (1997), Wong et al. (2002), de Koning et al. (2011), and Salle (2014). The given categories were collected, merged if necessary and refined based on the results of the pilot phase, including written and non-verbal utterances. Finally, all identified categories were grouped together to the category “self-explanation” given above.

(b) *Identification of adequate and inadequate self-explanations*. Based on the verbal data, it was decided if a coded self-explanation was adequate or inadequate. If a self-explanation could not be identified as either, it was left unclassified in step (b). The following category scheme was used:

• (*Adequate*): Self-explanations that matched the goals of the intervention phase were coded as adequate self-explanations, e.g. if a learner identified the vector *s* as geometrical representation of the symbolically given complex number *s* (list “Objects of the intervention” in section “Materials and Methods”).
• (*Inadequate*): With regard to the mathematical subject of complex numbers, self-explanations that revealed misconceptions, misunderstandings or inference errors were coded as inadequate, e.g. if a learner mistakenly explained the form of an equation by a multiplication with *i* instead of a rearrangement of terms.

(U*nclassified*): If it could not be derived whether the self-explanation was adequate or not, the self-explanation passage was labeled as unclassified.

Based on the mathematical content analysis described in Section “Materials and Methods,” misconceptions and typical errors were determined. The formulation of the category scheme for adequate and inadequate self-explanations was based on Wilkin (1997), McNamara (2004), Butcher (2006), Ainsworth and Burcham (2007), and de Koning et al. (2011) regarding the remarks in Section “Adequate and Inadequate Self-Explanations.” The categories of adequate and inadequate self-explanations were then supplemented with findings from the pilot phase.

### Second Coding Procedure – Verbal and Written Data

The second coding procedure followed the same steps as the first, but was based on the verbal data from the recordings supplemented by the written data. To synchronize the two data sources, the written data was scanned and segmented by time before the second coding procedure. This preliminary segmenting was conducted by a person not involved in the coding procedures. For every unit of analysis, it was checked, based on the written and video data, which written expressive products were present at its end and its beginning. By this comparison, it could be derived which annotations were made during that unit. The set of these annotations constituted the written data belonging to the respective unit of analysis.^9^

The two decisions described above (identification of self-explanations and identification of adequate and inadequate self-explanations) are supported by a broader data base which includes the intertwining of the two types of utterances (see theoretical framework). Based on these two data sources, coding was carried out simultaneously without knowing the results of the first procedure. Differences between the results of the two coding procedures (units of analysis that were coded as self-explanations in one of the two procedures but not the other) were analyzed again by a second person to avoid coding errors. Discrepancies were then solved by consensus.

### Third Coding Procedure – Verbal, Written and Non-verbal Data

For the third coding procedure, the data base was extended to verbal, written and non-verbal data. For every unit of analysis the coders listened to the audio data, read the segmented written products and, additionally, watched the video sequence of the participant’s body, hands, and all objects on the table in front of the participants.

Although the coding procedure applied here is not based on *transcriptions* of verbal and non-verbal data, methodological advices from McNeill (2005, 263f.) were considered for the coding of the video sequences. Before beginning the coding procedures, the first pass to “facilitate interpretations of gesture productions on later passes,” an analysis of the “product of elicitation” (results of the mathematical content analysis described in “Materials and Methods”), was discussed with all coders (McNeill, 2005, p. 264). Several purposes of the passes 5 and 6 described by Duncan and McNeill were considered for the coding procedure including non-verbal data to identify locations of gestures, beginning and ending of gesture phrases, and the identification of movements as gestures or actions. Hence, during the third coding procedure and based on the interplay of the three data sources, coders answered the following questions before they decided whether a unit of analysis could be counted as a self-explanation, respectively, as an adequate or inadequate self-explanation: is there a relevant movement visible in the non-verbal data? Is the relevant movement a gesture or an action? Is the whole gesture (“gesture phrase”) or action located inside the unit of analysis or does it begin/end in a adjacent unit? The respective answers provided the foundation for the categorization of passages as self-explanations and their classification as adequate or inadequate. Again, discrepancies in the three coding procedures as described above were analyzed by a different person to avoid errors and then solved by consensus.

### Inter-Coder Reliability

Inter-coder reliability for all coding procedures was ascertained by two coders based on categorizations of 10% of the data. Because of the small number of categories, all reliability values were calculated with Cohen’s κ, which takes random matches into account (Wirtz and Caspar, 2002). For the coding of self-explanations, the inter-coder reliability was 86.96% for the coding procedure based on verbal data, 88.93% for the procedure based on verbal and written data, and 89.2% for the coding based on all available data. Decisions on adequate and inadequate self-explanations showed an inter-coder reliability of 84.79% based on verbal data, 88.6% based on verbal and written data, and 90.11% for the coding based on all available data. Discrepancies were resolved by consensus.

## Results

The results section consists of four parts. Subsections “Coding of Self-Explanations,” “Changes of the Coding Results With Different Data Bases,” and “Alteration of the Interpretation of a Self-Explanation” depict results concerning the first research question (see section “Research Questions”): in what way will the consideration of non-verbal utterances (e.g. spontaneous gestures and actions) and written utterances (e.g. notes and diagrams) alter or support the coding of self-explanations? Subsection “Adequate and Inadequate Self-Explanations” depicts results concerning the second research question: In what way will the consideration of non-verbal and written utterances alter or support the determination of self-explanations as adequate or inadequate?

**FIGURE 2:**
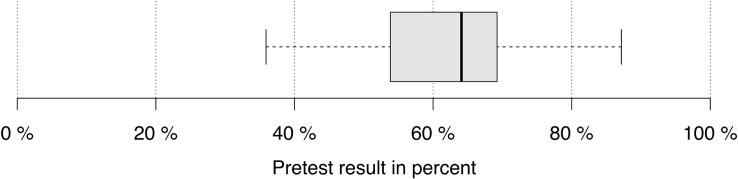
Pre-test results of all participants.

The pre-test results of the 33 subjects range between 36% and 87% (Figure 2). All participants had the basic knowledge that was necessary for the intervention phase (basic knowledge of sine and cosine, calculations with sine, cosine and real functions, and rearranging equations). There are small correlations between pre-test results and the number of coded self-explanations that increase with additional data sources (Table 1). Therefore, a strong relationship between prior knowledge and coding results could be ruled out.

**TABLE 1:** Correlations (Bravais–Pearson) between pre-test results and coding results.

	Verbal	Verbal and written	Verbal, written, and non-verbal
Pretest result in %	0.17	0.21	0.27

### Coding of Self-Explanations

**FIGURE 3:**
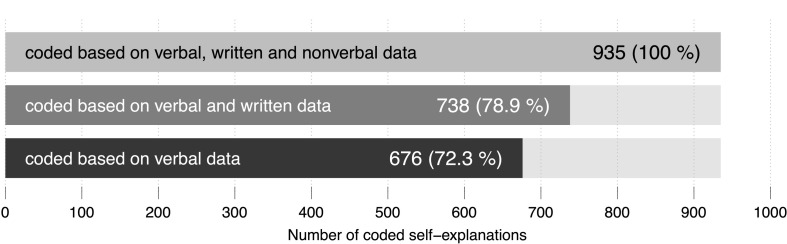
Numbers and percentages of self-explanations coded during the three coding procedures.

On average, each one of the 33 data sessions lasted about 24:36 min (standard deviation: 14:28 min). In total, 935 self-explanations were coded based on all available data. Without non-verbal data and based on verbal and written data only, 738 self-explanations were coded, which amounts to a difference of 197 self-explanations. From the 197 self-explanations 31 could be coded because of actions like using the calculator or the ‘cheat sheet.’ The remaining 166 could be coded because of gestures and their intertwining with verbal and written data. In all but three of these 166 passages, participants used gestures that point to or retrace objects depicted in the material. Based on verbal data only, 676 self-explanations were coded; this amounts to a difference of 62 self-explanations that were not coded because written data were not considered. Compared to the coding results based on all available data, 259 self-explanations were not coded based on verbal data (Figure 3).

**FIGURE 4:**
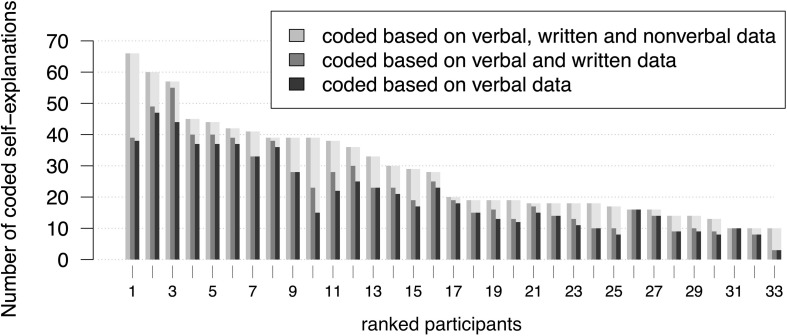
The lengths of the bars depict the individual numbers of self-explanations coded during the three coding procedures. Subjects were ranked with regard to their number of self-explanations that was coded based on verbal, written and non-verbal data.

The individual results of the self-explanation coding show differences in the number of self-explanations coded during each of the three coding procedures (Figure 4). Some participants’ self-explanations were coded based nearly exclusively on verbal data (no. 8, 16, 17, 26 in Figure 4), while others show more codings based on all available data (no. 1, 10, 11, 24, 25, 33 in Figure 4). Other learners showed greater numbers of self-explanations that were coded based on verbal and written data (no. 3, 10 in Figure 4).

The following sequence gives an example for a self-explanation that could only be coded during the coding procedure based on all available data.

**FIGURE 5:**
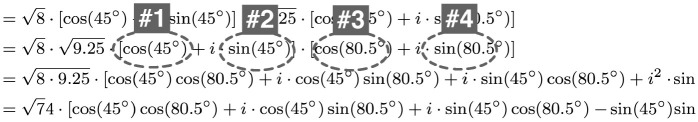
Snippet of Leo’s example sheet. Leo points at these four positions while she seems to read out parts of the line below.

*A self-explanation only coded due to the inclusion of non-verbal data*. Leo (participant no. 24 in Figure 4) is reading the lines of the calculation on the first example sheet (see Figure 5). The transcript depicts two units of analysis, divided by the pause in line 2.

Leo: At first cosine times cosine (points at #1 and #3) sine times co- (points at #2 and #3).,
ah, at first cosine times sine, (points at #1 and #4) sine times cosine (points at #2 and #3), and sine times sine (points at #2 and #4) with the i square.

Looking only at the verbal protocol without considering the accompanying pointing gestures, it seems as if Leo reads aloud the third line in the snippet, because the words refer almost exactly to the depicted formula (Figure 5). However, the accompanying pointing gestures intertwined with the verbal utterances reveal a self-explanation: She connects line two (the line she is pointing at) and line three (the line she compares her words to) of the calculation by carrying out the expansion of the product with her fingers. Hence, based on all available data, this segment could be coded as a self-explanation in the third coding procedure. During this scene, Leo did not produce written data. This example illustrates a behavior frequently observed during the coding procedures. Verbal utterances that seem to be read aloud text passages become self-explanations when taking accompanying gestures into account.

### Changes of the Coding Results With Different Data Bases

On average, 72.3% of all coded self-explanations were coded based on verbal data. Thus, there is an average difference of 28.14% in contrast to the coding based on all available data (see Figure 3). The individual differences between results based on these two data bases vary with a standard deviation of 16.19%. Individual results describing the proportion of self-explanations that were coded based on all available data range between 70% (learner no. 33 in Figure 4: seven out of ten self-explanations were coded based on all available data) and 0% (learners no. 26 and no. 31 in Figure 4: all self-explanations were coded based on verbal data only). Although there is a tendency toward higher differences in higher ranks, higher as well as lower differences can be found across the whole spectrum of ranks.

**FIGURE 6:**
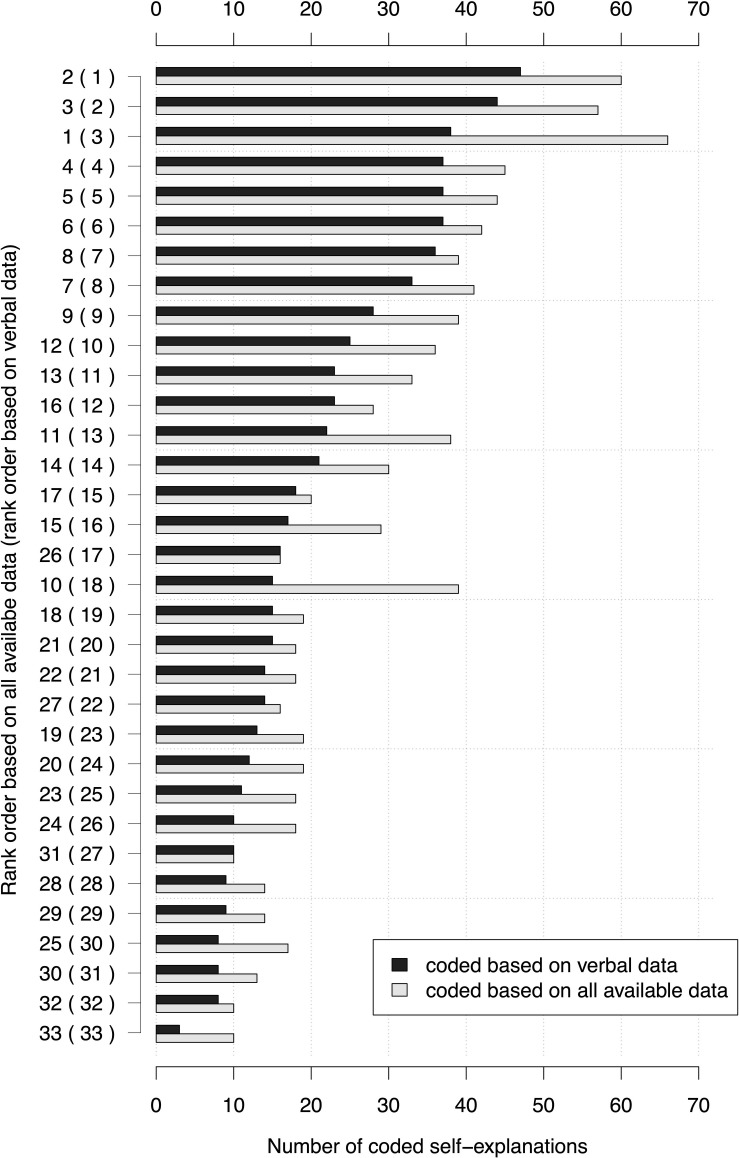
Results of coding self-explanations with different data bases. The black bars depict the number of coded self-explanations based on verbal data. The light gray bars depict the number of coded-self-explanations based on verbal, written and non-verbal data. Bars are ordered according to the numbers of self-explanations coded based on verbal data (numbers in parentheses). In comparison, the order according to the numbers of self-explanations coded based on verbal, written and non-verbal data is depicted.

Although some partial sequences remain the same, there is a non-negligible difference between the different ranks and the different total numbers of self-explanations that could affect, e.g. subsequent statistical calculations predicting individual achievement (see Figure 6).

### Alteration of the Interpretation of a Self-Explanation

**FIGURE 7:**
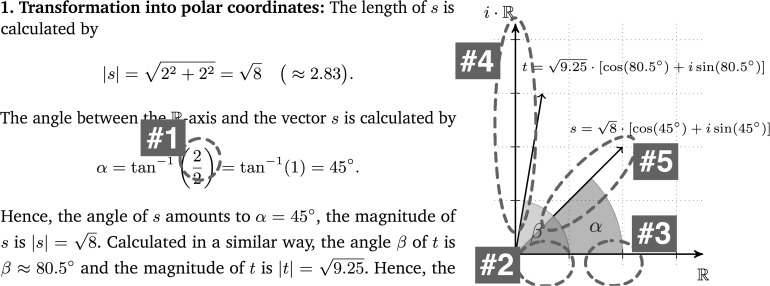
Snippet corresponding to Lena’s (#1, #2, #3) as well as Emily’s (#4, #5) protocols in Sections “Alteration of the Interpretation of a Self-Explanation” and “Adequate and Inadequate Self-Explanations.”

The quantitative coding does not consider the concrete interpretation of a self-explanation, e.g. whether a coded self-explanation is an activation of prior knowledge or the identification of a goal of an operation. However, a detailed analysis of certain self-explanations reveals a further aspect regarding the influence of written and non-verbal data on the identification of self-explanations. The following snippet shows in detail how a self-explanation receives an additional meaning when it is interpreted based on all available data. Lena (participant no. 10 in Figure 4) is working on the second example and tries to understand the transformation of $s=-\frac{1}{2}+\frac{1}{2}\cdot i$ into polar coordinates (see Figure 7).

Lena: Now I ask myself why, where this two by two comes from, (points at #1) but, .ah, exactly, this is here (points at #2) (points at #3). opposite side (lifts the pencil and points at #3 again). by adjacent side.

This self-explanation was coded based on verbal data because the words “opposite side” and “adjacent side” are neither depicted on the worked-out example nor on the ‘cheat sheet.’^10^ So, without considering the pointing gestures, this self- explanation would be described as an activation of prior knowledge because Lena uses the technical terms she has learned in class. However, considering the pointing gestures leads to an additional interpretation as an integration of geometric and symbolic representations, because the participant combines the fraction and coordinate system using her words and gestures simultaneously. Generally, the additional data often revealed integrations of representations (as can be seen in Lena’s protocol) or links between different information.

Results so far have focused on whether a unit of utterances is a self-explanation or not and how a self-explanation can be interpreted. In the following subsection, the influence of the different data on the categorization of self-explanations as adequate and inadequate will be analyzed.

### Adequate and Inadequate Self-Explanations

Of all 935 self-explanations, 835 could be determined as adequate and 68 as inadequate. In the end, 32 self-explanations could not be identified as adequate or inadequate and were left unclassified.

#### Number of Coded Adequate and Inadequate Self-Explanations

**FIGURE 8:**
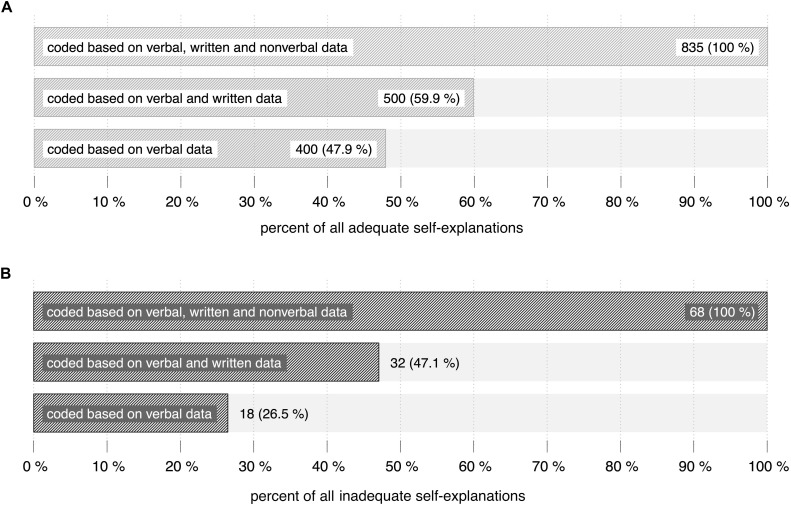
Data used to code adequate **(A)** and inadequate **(B)** self-explanations. The first row depicts the proportion based on verbal, written and non-verbal data. The second row depicts the corresponding proportion based on verbal and written data. The third row depicts the proportion of adequate self-explanations that were coded based on verbal data.

**FIGURE 9:**
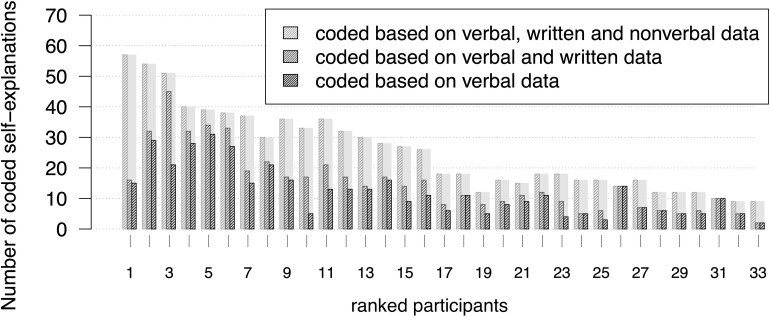
Data used to code adequate self-explanations. The subjects are ordered according to the number of self-explanations coded based on verbal, written, and non-verbal data (same order as in Figure 4).

835 adequate self-explanations could be identified by considering all available data. Based on verbal and written data, only 59.9% (500 of 835 self-explanations) were coded as adequate. About 48% (400 of 835 self-explanations) were coded as adequate based on verbal data (Figures 8A,B). Comparing the coding procedure based on verbal data and the coding procedure based on all available data, individual coding results of adequate self-explanations vary with a standard deviation of 20.55% around the mean of 48% (Figure 9). The individual proportion of adequate self-explanations coded based on all available data ranges between 84.8% (learner no. 10: only 5 of 33 adequate self-explanations were coded based on verbal data) and 0% (learners no. 26 and no. 31: all coded adequate self-explanations were coded based on verbal data). Thus again, although there is a tendency toward higher differences in higher ranks, higher differences as well as lower differences can be found across the whole spectrum of ranks. The overall percentages are even more distinct when reconstructing inadequate self-explanations: 68 inadequate self-explanations could be identified by considering all available data (*n* = 68). Based on verbal and written data, 47.1% (32) self-explanations were categorized as inadequate. Considering verbal data, only 26.5% (18) inadequate self-explanations could be identified.

To give an insight into the importance of non-verbal data for the coding of inadequate self-explanations, a typical example is presented in the following paragraph.

#### An Example of an Inadequate Self-Explanation Coded Based on Verbal and Non-verbal Data

Emily (participant no. 1 in Figure 4) reads out the fourth line of the solution: “The angle between the *R*-axis and the vector *s* is.” (Figure 7).

Emily: Ok, the angle between the R-axis,. that’s this one (traces along the i⋅R-axis at #4). and the vector s, this one. (traces along vector s at #5).

Without the pointing gestures, the coder does not know which part of the example her comment “that’s this one” refers to. From the intertwining of speech and gesture it can be recognized that she is identifying the wrong axis. In general, self-explanations like linking of concepts or notions to geometrical representations are often accompanied by written notes or pointing gestures. In order to decide whether a self-explanation is adequate or not, coders have to know the positions, terms and numbers in the given calculation to which participants may refer.

## Discussion

### Summary of the Findings

Although more than two-thirds of all self-explanations (676 of 935) could be reconstructed solely based on verbal data, the consideration of gestures, actions and written products notably affected individual results distinctly: individual differences in self-explanations coded in the three procedures vary independently of the amount of self-explanations and the resulting ranks. The results underline the key role of video data to link the instructional material and written notes to verbal utterances: taking into account more data results in more distinct changes in the ranking. An analysis including written and non-verbal data seems to fit better to the concept of self-explaining than an analysis without.

The influence of written and non-verbal data is even more important for the coding of self-explanations as adequate or inadequate. Verbal data often leaves the researcher uncertain with regard to objects referred to and connections emphasized by participants. Taking into account the intertwining of verbal utterances, written notes and gestures seems to improve the reconstruction of adequate and inadequate self-explaining and, hence, complex learning processes.

The variability of individual coding results for self-explanations as well as adequate and inadequate self-explanations may have several reasons. Important factors why people gesture more or less are the spoken language (Pika et al., 2006), differences in cognitive abilities (e.g. Hostetter and Alibali, 2007; Wartenburger et al., 2010) or other personal characteristics (Hostetter and Potthoff, 2012). Apart from general differences in gesture frequency, processing difficulties and the strength of mental representations may have an influence on how frequently learners use gestures (Melinger and Kita, 2007; Sassenberg and van der Meer, 2010). Another explanation for the differing coding results based on written data might be the differing use of learning strategies that need paper and pencil, like note-taking (Kiewra et al., 1995; Kobayashi, 2006). Last but not least, the general use of self-explaining activities influence the individual coding results as well; empirical studies show that students have different self-explanation-styles and, therefore, the quality and quantity of spontaneously generated self-explanations may differ distinctly (Renkl, 1997).

### Methodological Discussion

The analyses described in this paper tried to thoroughly investigate multimodality and self-explanations, which is a very effortful endeavor. In order to facilitate similar methodological approaches, e.g. the application of tablets could decrease the effort: participants’ written products could be segmented and assigned to specific units of analysis automatically. By omitting facial expressions, the current analysis of non-verbal data was less complex than an analysis including such expressions; however, considering such data could increase the precision of self-explanation analyses. The same holds true for gaze analysis via eye-tracking devices, which may give further insights into learners’ self-explaining activities (e.g. Conati and Merten, 2007; Hodds et al., 2014).

This paper follows a traditional approach to self-explanation analysis as applied in many previous studies (e.g. Chi et al., 1989; Renkl, 1997; McEldoon et al., 2013) and extends this approach by including further modalities. Hence, on the one hand, the constant segmentation used throughout the study provides a reliable frame for a one-to-one comparison of analyses considering different data bases. Furthermore, the results offer some perspectives for methodological variations of traditional self-explanation studies. For example, by consideration of written and non-verbal data, maybe even sparse verbal protocols may reveal self-explaining activities (such sparse protocols were mentioned, e.g. in Renkl, 2002; Renkl et al., 2004). On the other hand, certain characteristics of multimodality could not be considered in the analysis, e.g. in what way different modalities alter the segmentation of the data or whether there are fundamental differences between self-explanations accompanied by gestures or actions and self-explanations unaccompanied by gestures or actions. The use of gestures could be related to the proximity of uttered concepts to practical actions (Kita et al., 2017) or to the use of metaphors that express spatial concepts (Lakoff and Johnson, 1980; Lakoff and Núñez, 2000). Other explanations may refer to the cognitive effort – the more demanding cognitive processes are, e.g. conceptualization, the more often gestures occur (Hostetter et al., 2008) – or to the durability of self-explanations: frequent gesturing will “make learning last” (Wagner Cook et al., 2008).

The fact that in the present study 676 self-explanations could be coded solely based on verbal data does not imply the absence of gestures. Although not necessary for the coding decision, more than two thirds of these 676 self-explanations and about 80% of all 935 coded self-explanations were accompanied by gestures or actions.^11^ Thinking aloud appears to be strongly connected to gesturing and acting in silence (Schwartz and Black, 1996; Hegarty et al., 2005), particularly if no visible human person is available as a dialogue partner (Emmorey and Casey, 2001).

More detailed analyses regarding the interplay of and emphasis on the modalities involved could provide deeper insights into the learners’ cognitive effort of learners (e.g. more frequent use of gestures when learners engage in more demanding cognitive processes), their specific use of gestures in combination with speech and writing (e.g. lowering cognitive load by locating things with gesture or writing things down; intertwining use of writing and gesturing for themselves) and, more generally, into their generative cognitive activities. In combination with the broadened concept of self-explanation introduced in this paper, answers to these questions may provide a foundation for an intensified conceptual discussion and for the integration of self-explanations and gestures (Alibali and Goldin-Meadow, 1993; Kita et al., 2017). Comparisons to gesture-speech mismatches (Alibali and Goldin-Meadow, 1993), growth points (McNeill, 2002) or, more general, cognitive functions of gesturing like the activation, the manipulation, the packaging or the exploration of spatio-motoric information (Kita et al., 2017) offer promising starting points for further theoretical integration.

### Implications for Learning and Instruction

Since instructional learning is an important method in schools and universities around the world, the implementation of self-explaining in such settings is highly relevant (Chiu and Chi, 2014). Considering the results presented in this paper, teachers seeking to identify specific self-explaining processes applied by learners have to carefully consider not only verbal utterances, but all multimodal aspects. Especially the subtle changes expressed by writing, gesturing and their combined use can help determine concrete self-explaining processes carried out more precisely (e.g. distinguishing reading of a passage, activating prior knowledge and integrating different representations) in order to facilitate individual learning processes. Based on the qualitative analyses in this study, it could also be hypothesized that a classification of high quality and low quality self-explanations would be affected distinctly by the consideration of more modalities (cf. Roy and Chi, 2005). This could be of some benefit especially to the identification of successful self-explaining activities and elicitations through self-explanation prompts or trainings in the classroom, and could hereby confirm or alter the scope of such interventions to foster meaningful learning (Chi et al., 1994; Berthold et al., 2009).

Furthermore, teachers often support their explanations by writing, diagrams on a blackboard or a slideshow, and gestures (Alibali et al., 2014; Yeo et al., 2017). Since these teachers’ gestures influence the learners’ understanding of learners and are partly imitated by them (e.g. Goldin-Meadow et al., 1999; Cook and Goldin-Meadow, 2006), teachers’ awareness of how such gestures influence learners’ explanations and self-explaining could lead to a deeper understanding of what’s going on in the learners’ minds. Additionally, for learners as well as teachers, knowing in what way gesturing (to oneself and to others) can influence self-explaining would be of extremely high value for the understanding and improvement of learning. Deeper analyses of the functions and purposes of gestures during self-explaining could, for example, clarify to what extent actions and gestures may decrease cognitive load during learning (Goldin-Meadow, 2010; Alibali et al., 2011; Krause and Salle, 2016; Kita et al., 2017). This information could provide learners with the opportunity to optimize their self-explaining by an adequate use of specific gesturing.

### Implications for Further Research

Several questions arise from the results presented. This paper does not address the influence of different coding results on predictions of achievement; to confirm the predictive power of multimodal self-explanations, further studies may quantify the extent to which those predictions vary by the inclusion of additional data sources during analysis. Therefore, multimodal self-explanations may serve as reliable measures for the construction and evaluation of instructional materials (e.g. Renkl, 2005; Butcher, 2006; de Koning et al., 2011). Furthermore, future analyses may reveal the extent to which gestures accompanying self-explanations can be characterized in terms of iconic, metaphorical and deictic gestures (McNeill, 1992; Goodwin, 2003; Edwards, 2008), or, in more detail, as tracing gestures or pointing gestures (cf. Hegarty et al., 2005). Additionally, it remains unclear to what extent these gestures can be found when dealing with geometrical representations instead of symbolic calculations, in what way they are intertwined with simultaneous verbal and written utterances and whether the presence or absence of the learning material and the presentation on paper or screen influence gesturing and learning. Regarding current theories on cognitive functions of gestures, answers to these questions could shed light on more fundamental questions regarding the use of gestures during thinking (Kita et al., 2017).

Although the results discussed here are specific to the mathematical domain, it could be hypothesized that written and non-verbal data may influence the results of self-explanation studies in other domains, too. The analytical approach therefore may provide a promising starting point for a deeper analysis of cognitive activities in general.

## Data Availability Statement

The datasets generated for this study are available on request to the corresponding author.

## Ethics Statement

The patients/participants provided their written informed consent to participate in this study, which satisfied the ethical criteria and dispositions of our institution.

## Author Contributions

The author confirms being the sole contributor of this work and has approved it for publication.

## Conflict of Interest

The author declares that the research was conducted in the absence of any commercial or financial relationships that could be construed as a potential conflict of interest.

## Footnotes

1 Studies reviewed: Chi et al. (1989, 1994), Chi and VanLehn (1991), Pirolli and Recker (1994), Bielaczyc et al. (1995), Recker and Pirolli (1995), Renkl (1997, 2002), Neuman and Schwarz (1998), Renkl et al. (1998), Renkl et al. (2004), Stark (1999), Neuman et al. (2000), Wong et al. (2002), Ainsworth and Loizou (2003), McNamara (2004), Butcher (2006), Ainsworth and Burcham (2007), de Koning et al. (2011), Durkin and Rittle-Johnson (2012), Lin and Atkinson (2013), McEldoon et al. (2013), and Rittle-Johnson et al. (2015).

2 An exception is Stark (1999) who applied a “live coding” during the intervention phase.

3 Although some studies (e.g. Chi et al., 1989) allowed drawing and notetaking, no hints could be found that these documents were considered in the self-explanation data analysis.

4 A language ‘spoken’ through specific gestures (Sfard, 2008).

5 The worked-out examples were constructed based on state-of-the-art guidelines for instructional material (e.g. Mayer, 2014).

6 This mathematical content analysis was carried out by a mathematician and two researchers of mathematics education, one of them being the author.

7 A list summarizing necessary prior knowledge was another result of the mathematical content analysis mentioned above.

8 One student was excluded.

9 This comparison was based on video data (see also the discussion section).

10 In the original protocol, she uses the words “Gegenkathete” for opposite side and “Ankathete” for adjacent side, which are technical terms in German.

11 Details of this analysis are not depicted in this paper.

## Supplementary Material

Supplementary Data Sheet 1 List of self-explanations.

Supplementary Data Sheet 2 Worked-out examples.
