# Supplementary material for: Analyzing Self-Explanations in Mathematics: Gestures and Written Notes Do Matter
Source: Front Psychol. 2020 Nov 23;11:513758. doi: 10.3389/fpsyg.2020.513758 (PMC7720934; doi:10.3389/fpsyg.2020.513758)
Supplement: Supplementary Data Sheet 1 — List of self-explanations. [file Data_Sheet_1.pdf]

List of self-explanations (first worked-out example)

(1) … explains the necessity of knowing the vector's length and the angle alpha for converting the complex numbers.

(1) … explains that the real part of s and t is represented as place on the x-axis and the imaginary part of s and t as place on the y-axis.

(1) … identifies the vector s, given in the algebraic form, in the geometrical representation as the lower arrow as well as the vector t as the second, higher arrow.

(1) ... explains the calculation of the vector's length as application of the Pythagorean Theorem.

(1) … recognizes the Cartesian coordinates and their representation on the axis of coordinates.

(1) … recognizes the calculation of the angle α as the application of a trigonometric equation in a right-angled triangle

(1) … recognizes that the angle β in the coordinate system is 80,5°.

(1) … identifies that length and magnitude of a vector are equal.

(1) … explains that the polar coordinates contain the previously calculated length of the vector s and the angle α respectively the vector t and the angle β.

(1) … integrates the symbolic representation of a complex number and its parts with respective characteristics of the geometrical counterpart.

(2) … identifies the product in line two as a multiplication of the previously calculated polar coordinates of s and t.

(2) … recognizes the possibility of changing the factors by using the commutative property from line two to three.

(2) … explains the change from line three to four of the calculation by the distributive property.

(2) … recognizes the use of the properties of real roots and rules for operations with surds involving positive radicands from line three to four.

(2) … explains the change of the algebraic sign between line four and five of the calculation by the relation $i^2 = 1$.

(2) … identifies the factor $\sqrt{74}$ as a simplification of $\sqrt{8 \cdot 9,25}$ from line four to five

(2) … explains the simplification from line five to six as the use of the commutative property of addition.

(2) … explains that rearranging the sum and the use of the distributive property from line five to six leads to a representation as polar coordinates in line seven.

(2) … explains the simplification from line six to seven of the calculation as an application of the addition theorems.

(2) … recognizes the symbolic term in line seven as a new complex number and therefore as result of the calculation of $s \cdot t$ before.

(2) … recognizes that the goal of all depicted calculation steps is a complex number represented as polar coordinates

(2) … recognizes the approximation of $\sqrt{74}$ in parentheses in line eight

(2) … recognizes the proper addition of 45° and 80,5° in line eight

(3) … integrates both graphical representations of s and t.

(3) … identifies the arrow on the left as a representation of the previously calculated vector $s \cdot t$.

(3) … recognizes the x-axis and the y-axis of coordinates as real and imaginary axis for polar coordinates.

(3) … identifies the factor $\sqrt{74}$ as the length of the vector $s \cdot t$.

(3) … recognizes that the resulting angle γ of $s \cdot t$ is the sum of α and β.

(3) … recognizes that the resulting length of $s \cdot t$ is the product of $|s|$ and $|t|$.
