# Supplementary material for: Analyzing Self-Explanations in Mathematics: Gestures and Written Notes Do Matter
Source: Front Psychol. 2020 Nov 23;11:513758. doi: 10.3389/fpsyg.2020.513758 (PMC7720934; doi:10.3389/fpsyg.2020.513758)

Task 1: Transform $s = 2 + 2i$ and $t = 0.5 + 3i$ into polar coordinates, multiply the complex numbers and plot s, t and $s \cdot t$ into a coordinate system.

Solution:

1. Transformation into polar coordinates: The length of s is calculated by

$$|s| = \sqrt{2^2 + 2^2} = \sqrt{8} \quad \big(\approx 2.83 \big).$$

The angle between the $\mathbb{R}$-axis and the vector s is calculated by

$$\alpha = \tan^{-1}\left(\frac{2}{2}\right) = \tan^{-1}(1) = 45^\circ.$$

Hence, the angle α amounts to $\alpha = 45^\circ$, the magnitude of s is $|s| = \sqrt{8}$. Calculated in a similar way, the angle β of t is $\beta \approx 80.5^\circ$ and the magnitude of t is $|t| = \sqrt{9.25}$. Hence, the polar coordinates are:

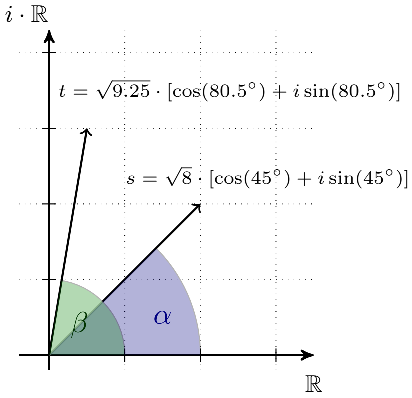

$$s = \sqrt{8} \cdot [\cos(45^\circ) + i \cdot \sin(45^\circ)] \qquad \text{und} \qquad t = \sqrt{9.25} \cdot [\cos(80.5^\circ) + i \cdot \sin(80.5^\circ)]$$

2. Multiply the complex numbers in polar coordinates:

$$\begin{aligned}
s \cdot t &= |s| \cdot (\cos\alpha + i \cdot \sin\alpha) \cdot |t| \cdot (\cos\beta + i \cdot \sin\beta) \\
&= \sqrt{8} \cdot [\cos(45^\circ) + i \cdot \sin(45^\circ)] \cdot \sqrt{9.25} \cdot [\cos(80.5^\circ) + i \cdot \sin(80.5^\circ)] \\
&= \sqrt{8} \cdot \sqrt{9.25} \cdot [\cos(45^\circ) + i \cdot \sin(45^\circ)] \cdot [\cos(80.5^\circ) + i \cdot \sin(80.5^\circ)] \\
&= \sqrt{8 \cdot 9.25} \cdot [\cos(45^\circ)\cos(80.5^\circ) + i \cdot \cos(45^\circ)\sin(80.5^\circ) + i \cdot \sin(45^\circ)\cos(80.5^\circ) + i^2 \cdot \sin(45^\circ)\sin(80.5^\circ)] \\
&= \sqrt{74} \cdot [\cos(45^\circ)\cos(80.5^\circ) + i \cdot \cos(45^\circ)\sin(80.5^\circ) + i \cdot \sin(45^\circ)\cos(80.5^\circ) - \sin(45^\circ)\sin(80.5^\circ)] \\
&= \sqrt{74} \cdot [\cos(45^\circ)\cos(80.5^\circ) - \sin(45^\circ)\sin(80.5^\circ) + i \cdot [\cos(45^\circ)\sin(80.5^\circ) + \sin(45^\circ)\cos(80.5^\circ)]] \\
&= \sqrt{74} \cdot [\cos(45^\circ + 80.5^\circ) + i \cdot \sin(45^\circ + 80.5^\circ)] \\
&\big(\approx 8.602 \cdot [\cos(125.5^\circ) + i \cdot \sin(125.5^\circ)] \ \big)
\end{aligned}$$

3. Geometrical representation:

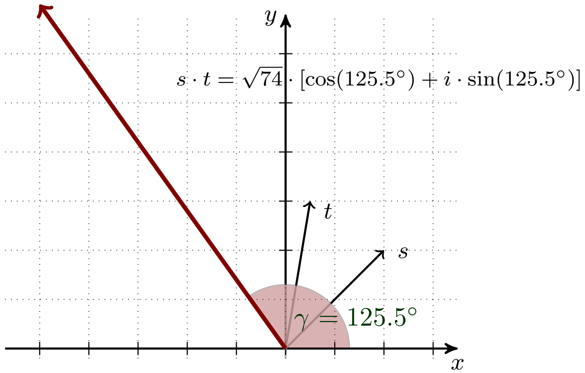

Task 2: Transform $s = -\frac{1}{2} + \frac{1}{2}i$ and $t = -\frac{1}{2} - \frac{1}{2}i$ into polar coordinates, multiply the complex numbers and plot s, t and $s \cdot t$ into a coordinate system.

Solution:

1. Transformation into polar coordinates: The length of s is calculated by

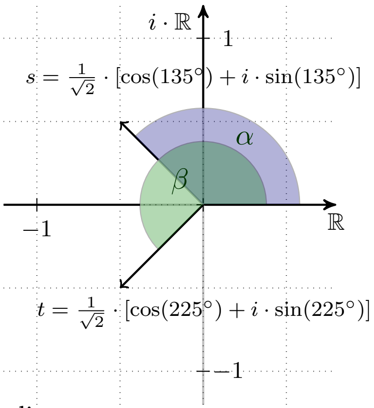

$$|s| = \sqrt{\left(\frac{1}{2}\right)^2 + \left(\frac{1}{2}\right)^2} = \sqrt{\frac{1}{4} + \frac{1}{4}} = \sqrt{\frac{1}{2}} = \frac{1}{\sqrt{2}} \quad (\approx 0.71).$$

The angle between the $\mathbb{R}$-axis and the vector s is calculated by

$$\alpha = \tan^{-1}\left(\frac{\frac{1}{2}}{-\frac{1}{2}}\right) = \tan^{-1}(-1) = 135°.$$

Hence, the angle α amounts to $\alpha = 135°$, the magnitude of s is $|s| = \frac{1}{\sqrt{2}}$. Calculated in a similar way, the angle β of t is $\beta = 225°$ and the magnitude of t is $|t| = \frac{1}{\sqrt{2}}$. Hence, the polar coordinates are:

$$s = \frac{1}{\sqrt{2}} \cdot [\cos(135°) + i \cdot \sin(135°)] \qquad \text{und} \qquad t = \frac{1}{\sqrt{2}} \cdot [\cos(225°) + i \cdot \sin(225°)]$$

2. Multiply the complex numbers in polar coordinates:

$$\begin{aligned}
s \cdot t &= \frac{1}{\sqrt{2}} \cdot [\cos(135°) + i \cdot \sin(135°)] \cdot \frac{1}{\sqrt{2}} \cdot [\cos(225°) + i \cdot \sin(225°)] \\
&= \frac{1}{\sqrt{2}} \cdot \frac{1}{\sqrt{2}} \cdot [\cos(135°) + i \cdot \sin(135°)] \cdot [\cos(225°) + i \cdot \sin(225°)] \\
&= \frac{1}{\sqrt{2} \cdot \sqrt{2}} \cdot [\cos(135°)\cos(225°) + i \cdot \cos(135°)\sin(225°) + i \cdot \sin(135°)\cos(225°) + i^2 \cdot \sin(135°)\sin(225°)] \\
&= \frac{1}{2} \cdot [\cos(135°)\cos(225°) + i \cdot \cos(135°)\sin(225°) + i \cdot \sin(135°)\cos(225°) - \sin(135°)\sin(225°)] \\
&= \frac{1}{2} \cdot [\cos(135°)\cos(225°) - \sin(135°) \cdot \sin(225°) + i \cdot [\cos(135°)\sin(225°) + \sin(135°) \cdot \cos(225°)]] \\
&= \frac{1}{2} \cdot [\cos(135° + 225°) + i\sin(135° + 225°)] = \frac{1}{2} \cdot [\cos(360°) + i\sin(360°)]
\end{aligned}$$

3. Geometrical representation:

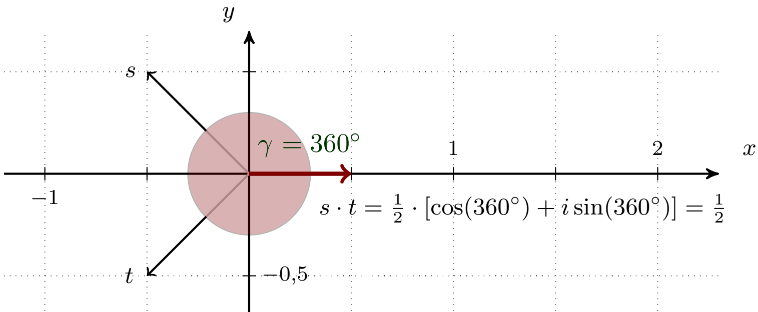

Aufgabe 3: Transform $a = 3 \cdot [\cos(30^\circ) + i \cdot \sin(30^\circ)]$ and $b = 2 \cdot [\cos(120^\circ) + i \cdot \sin(120^\circ)]$ into Cartesian coordinates, multiply the complex numbers and plot a, b und $a \cdot b$ into a coordinate system.

Solution:

1. Transformation into Cartesian coordinates:

$$
\begin{aligned}
a &= 3 \cdot [\cos(30^\circ) + i \cdot \sin(30^\circ)] \\
&= 3 \cdot [\frac{\sqrt{3}}{2} + i \cdot \frac{1}{2}] \\
&= \frac{3\sqrt{3}}{2} + i \cdot \frac{3}{2} \\
&(\approx 2.6 + i \cdot 1.5)
\end{aligned}
$$

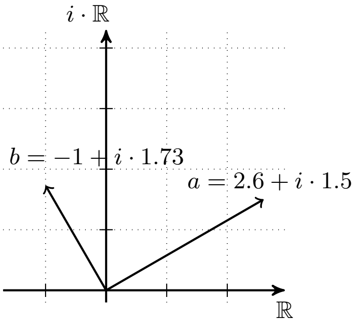

b is transformed in a similar way:

$$b = 2 \cdot [\cos(120^\circ) + i \cdot \sin(120^\circ)] = 2 \cdot [-\frac{1}{2} + i \cdot \frac{\sqrt{3}}{2}] = -1 + i \cdot \sqrt{3} \quad (\approx -1 + i \cdot 1.73)$$

Hence, the Cartesian coordinates of a und b are

$$a = \frac{3\sqrt{3}}{2} + i \cdot \frac{3}{2} \qquad \text{und} \qquad b = -1 + i \cdot \sqrt{3}.$$

2. Multiply the complex numbers in Cartesian coordinates:

$$
\begin{aligned}
a \cdot b &= \left(\frac{3\sqrt{3}}{2} + i \cdot \frac{3}{2}\right) \cdot \left(-1 + i \cdot \sqrt{3}\right) \\
&= \frac{3\sqrt{3}}{2} \cdot (-1) + \frac{3\sqrt{3}}{2} \cdot i \cdot \sqrt{3} + i \cdot \frac{3}{2} \cdot (-1) + i \cdot \frac{3}{2} \cdot i \cdot \sqrt{3} \\
&= -\frac{3\sqrt{3}}{2} + i \cdot \frac{9}{2} - i \cdot \frac{3}{2} + i^2 \cdot \frac{3\sqrt{3}}{2} \\
&= -\frac{3\sqrt{3}}{2} + i \cdot \left(\frac{9}{2} - \frac{3}{2}\right) - \frac{3\sqrt{3}}{2} \\
&= -\frac{6\sqrt{3}}{2} + i \cdot \frac{6}{2} = -3\sqrt{3} + i \cdot 3 \quad (\approx -5.2 + i \cdot 3)
\end{aligned}
$$

3. Geometrical representation:

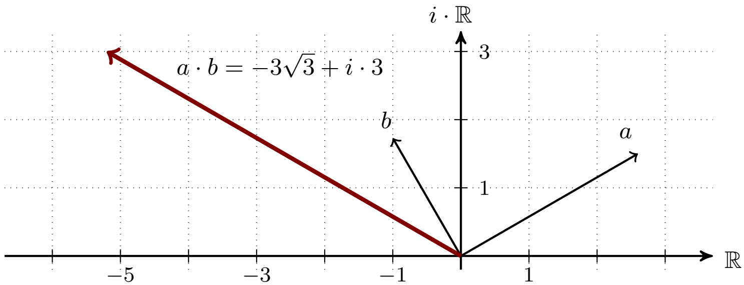

Supplement: Supplementary Data Sheet 2 — Worked-out examples. [file Data_Sheet_2.pdf]
